# Life-threatening subdural hematoma after aortic valve replacement in a patient with Heyde syndrome: a case report

**DOI:** 10.1186/s13019-017-0629-x

**Published:** 2017-08-08

**Authors:** Tetsuro Uchida, Azumi Hamasaki, Eiichi Ohba, Atsushi Yamashita, Jun Hayashi, Mitsuaki Sadahiro

**Affiliations:** Second Department of Surgery, Faculty of Medicine, Yamagata University, 2-2-2 Iida-Nishi, Yamagata, 990-9585 Japan

**Keywords:** Case report, Subdural hematoma, Aortic valve replacement, Heyde syndrome

## Abstract

**Background:**

Heyde syndrome is known as a triad of calcific aortic stenosis, anemia due to gastrointestinal bleeding from angiodysplasia, and acquired type 2A von Willebrand disease. This acquired hemorrhagic disorder is characterized by the loss of the large von Willebrand factor multimers due to the shear stress across the diseased aortic valve. The most frequently observed type of bleeding in these patients is mucosal or skin bleeding, such as epistaxis, followed by gastrointestinal bleeding. On the other hand, intracranial hemorrhage complicating Heyde syndrome is extremely rare.

**Case presentation:**

A 77-year-old woman presented to our hospital with severe aortic stenosis and severe anemia due to gastrointestinal bleeding and was diagnosed with Heyde syndrome. Although aortic valve replacement was performed without recurrent gastrointestinal bleeding, postoperative life-threatening acute subdural hematoma occurred with a marked midline shift. Despite prompt surgical evacuation of the hematoma, she did not recover consciousness and she died 1 month after the operation.

**Conclusions:**

Postoperative subdural hematoma is rare, but it should be kept in mind as a devastating hemorrhagic complication, especially in patients with Heyde syndrome.

## Background

Heyde syndrome is a complex disorder resulting from the association between calcific aortic stenosis (AS), gastrointestinal (GI) bleeding from angiodysplasia, and acquired type 2A von Willebrand disease [1–3]. Degradation of large von Willebrand factor (vWF) multimers by the shear stress across the diseased aortic valve plays a pivotal role for the coagulopathy in this syndrome [3]. Aortic valve replacement (AVR) is the first line therapy for patients with severe AS but can also be an effective treatment for bleeding angiodysplasia and acquired von Willebrand disease [4]. The use of cardiopulmonary bypass itself carries a high risk of postoperative bleeding [5]; hence, cardiac surgeons should pay attention to avoid recurrent GI bleeding and bleeding from other sites, such as epistaxis, ecchymosis, metrorrhagia, hematuria, and gingivorrhagia [3]. On the other hand, intracranial hemorrhage complicating Heyde syndrome has not been previously reported. Herein we describe an extremely rare case of Heyde syndrome associated with life-threatening postoperative acute subdural hematoma owing to the hemorrhagic element of the syndrome.

## Case presentation

A 77-year-old woman was admitted to a neighboring hospital presenting with severe anemia and increasing exertional dyspnea. Her hemoglobin level was found to be 6.1 g/dL. Thereafter, she had been receiving medical treatment for congestive heart failure due to severe AS and was receiving repeated blood transfusion for recurrent anemia. GI bleeding was considered as a possible cause of the severe anemia and she was referred to the Gastroenterology Department in our hospital. Upper and lower GI endoscopies revealed angiodysplasia in the transverse colon with visible acute bleeding. Endoscopic hemostasis was successfully performed using hemoclips.

**Fig. 1:**
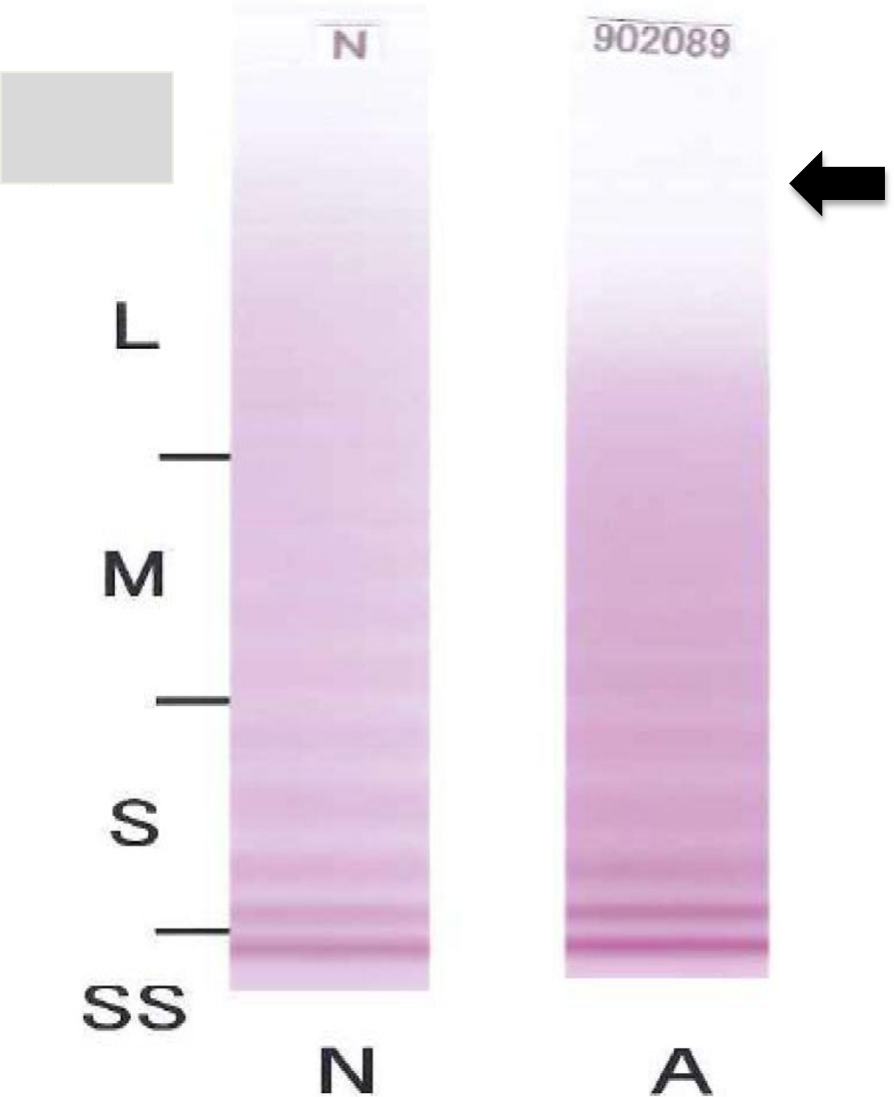
Multimer analysis of von Willebrand factor using gel electrophoresis showing the loss of high molecular weight multimers (*arrow*) in the plasma of the patient with Heyde syndrome. (L = Large multimer, M = medium multimer, S = small multimer, SS = smallest multimer, N = control, A = this patient)

During hospitalization echocardiography showed severe AS, with a peak aortic pressure gradient of 100 mmHg, and left ventricular ejection fraction of 70%. Although laboratory examinations confirmed mild anemia (hemoglobin level, 10.5 g/dL) after endoscopic hemostasis, a more subtle bleeding disorder could not be detected using global anticoagulation tests. The activity of vWF was normal (155%), but further analysis using gel electrophoresis revealed loss of large vWF multimers, which was a typical finding of acquired type 2A von Willebrand disease (Fig. 1). The diagnosis of Heyde syndrome was established and aortic valve replacement was planned on an elective basis. A brain computed tomography (CT) performed as a routine preoperative examination 1 week preoperatively showed no abnormal findings except for mild global cortical atrophy. She also had no previous head injuries.

Under general anesthesia, the patient underwent surgery through a median sternotomy. Cardiopulmonary bypass was established with ascending aorta and bicaval cannulation. Anticoagulation during cardiopulmonary bypass was maintained with the use of heparin to a target activated clotting time (ACT) of 300 to 400 s. After aortic cross clamping, cardiac arrest was achieved with a cardioplegic solution. The aortic valve was tricuspid and severely calcified. The valve and the calcified nodule were excised, and a 21-mm SOLO SMART stent-less bovine pericardial valve (Sorin Group, Saluggia, Italy) was implanted. Weaning from cardiopulmonary bypass was uneventful and no abnormal high ACT was recognized. Continuous real-time monitoring of regional oxygen saturation in the brain revealed no abnormalities throughout the operation.

**Fig. 2:**
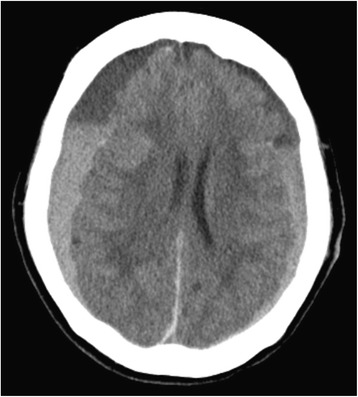
Brain computed tomography on the operative day. A large right subdural hematoma with secondary severe midline shift was observed

Postoperative hemodynamics were stable, but bilateral dilated pupils and loss of light reflex were noticed by an intensivist soon after intensive care unit admission. Her level of consciousness deteriorated to a Glasgow Coma Scale score of 3 and a Japan Coma Scale of III-300. An emergent brain CT revealed a large right subdural hematoma with severe midline shift (Fig. 2). In spite of her poor clinical condition, the decision of immediate surgical drainage was made by the neurosurgeons. Despite prompt surgical evacuation of the hematoma, her neurological condition did not improve. The volume drained from the chest tube was not significant. Recurrent GI bleeding was not observed during the postoperative period. She did not regain consciousness at all and she died 31 days after the cardiac operation.

## Discussion and conclusions

The association between AS and GI bleeding was first described by Heyde in 1958 [1]. In 1992, a combination of AS and recurrent GI bleeding from angiodysplasia was defined as Heyde syndrome [2]. Although the exact pathophysiology of the syndrome is still unclear, a recent report by Vincentelli et al. showed that acquired type 2A von Willebrand disease causes Heyde syndrome [3]. vWF synthesized by endothelial cells is stored as ultra-large multimers and mediates platelet adhesion for hemostasis. Degenerative AS is associated with increased destruction of high molecular weight multimers of vWF by the shear stress across the stenotic aortic valve. Degradation of the heaviest multimers of vWF, that can induce bleeding from intestinal angiodysplasia, is the basis for the coagulopathy in Heyde syndrome [3]. Heyde syndrome refers to a triad of aortic stenosis, acquired coagulopathy (type 2A von Willebrand disease) and anemia due to bleeding from intestinal angiodysplasia or from an idiopathic site [6]. All three criteria were positive in our patient.

Patients with Heyde syndrome who are treated by intestinal resection generally continue to bleed from other sites, while AVR usually cures the clotting disorder and the recurrent anemia [6]. AVR, rather than bowel resection, was considered to lead to long-term resolution of congestive heart failure and anemia in severe cases.

Intracranial hemorrhagic events occurring following open-heart surgery are uncommon but may prove devastating due to enhanced bleeding tendency caused by heparin administration, hypothermia, and postoperative anticoagulation therapy [7–10]. Of these, the incidence of subdural hematoma after open-heart surgery was 0.1% [7]. Subdural hematomas result from tearing of bridging veins between the dura and cerebral hemispheres, usually resulting from apparently trivial head injury. Krous et al. described the cause of subdural hematoma after cardiac surgery as a tearing of bridging veins that occurs with a fluid shift to the brain and a bleeding tendency induced by heparin administration [8]. Our patient had no history of head injury and a preoperative brain CT revealed no abnormalities. We suspected that the subdural hematoma may have been caused by rapid alterations in cerebral volume, leading to a tearing of the dural bridging veins under cardiopulmonary bypass. Once bleeding occurred, the subdural hematoma would rapidly enlarge owing to a bleeding tendency following postoperative consumptive coagulopathy and heparin administration. The hemorrhagic disorder associated with Heyde syndrome would enhance massive bleeding which resulted in the formation of the large subdural hematoma with severe midline shift. Subdural hematoma following open-heart surgery is sometimes life-threatening, but early diagnosis and prompt treatment may lead to neurological recovery in many cases [7–10]. Especially in our patient, severe hemorrhagic tendency enhanced by Heyde syndrome led to large subdural hematoma and irreversible neurological deterioration. Unfortunately, her neurological state did not improve and she died 1 month after the cardiac operation. Pre-existing coagulopathy, including Heyde syndrome, in patients undergoing open-heart surgeries may cause prolonged bleeding from the surgical sites or unexpected organ bleeding because of the necessity for heparinization. In conclusion, if patients with AS show GI bleeding, Heyde syndrome should be suspected, and vWF multimers should be evaluated for diagnosis [5]. Subdural hematoma subsequent to open-heart surgery is rare, but our experience may draw the attention of cardiac surgeons, not only to recurrent GI bleeding, but also to the possibility of acute subdural hematoma in patients with Heyde syndrome undergoing AVR. To our knowledge, this is the first report of life-threatening subdural hematoma complicating Heyde syndrome.

## Abbreviations

AS: Aortic stenosis
AVR: Aortic valve replacement
CT: Computed tomography
GI: Gastrointestinal
vWF: von Willebrand factor
